# Editorial: Rising stars: cell and stem cell transplantation 2022

**DOI:** 10.3389/frtra.2024.1356546

**Published:** 2024-01-23

**Authors:** Raffaella Greco, Maria Teresa Lupo Stanghellini

**Affiliations:** Unit of Hematology and Bone Marrow Transplantation, IRCCS San Raffaele Hospital, Vita-Salute San Raffaele University, Milan, Italy

**Keywords:** HSCT, CAR-T cells, GvHD, infections, VOD

**Editorial on the Research Topic** Rising stars: cell and stem cell transplantation 2022

The Research Topic “Rising Stars in Cell and Stem Cell Transplantation” includes authors from different countries and serves as a forum for new directions and emerging trends in the field of hematopoietic stem cell transplantation (HSCT) and cellular therapies.

This Research Topic includes a heterogeneous set of topics, spanning from allogeneic HSCT to chimeric antigen receptor (CAR) T cells, and potential complications such as graft versus host disease (GvHD), infections and hepatic veno-occlusive disease (VOD).

The Research Topic starts with the brief research report from El Cheikh et al., describing the experience of a transplant center in Lebanon with haploidentical HSCT for hematological malignancies in adult patients.

Numbers of HSCT in Europe and globally is rising, thanks to the massive expansion of transplant technology, including the notable developments in haploidentical HSCT with rapid donor availability (1). Moreover, the use of post-transplant cyclophosphamide (PT-Cy) (2) and reduced intensity conditioning (RIC) regimens (3) improved outcomes, expanded transplant procedure to frail/older candidates and decreased toxicities. In this paper, they reported encouraging results with haploidentical HSCT and PT-Cy as GvHD prophylaxis, confirming the feasibility of such approaches also in the Middle East and encouraging its implementation to centers across different countries.

The next manuscript reported the successful use of CAR-T cell therapy despite bacterial, fungal and Coronavirus disease 2019 (COVID-19) infections. Patients undergoing CAR-T cell therapy are often profoundly immunosuppressed from prior treatments and by concomitant hypogammaglobulinemia. These factors may increase the overall infection risk following cell infusion (4). Radici et al. reported the case report of a patient affected by refractory mantle cell lymphoma achieving complete metabolic remission after Brexucel. During the treatment course, the patient experienced multiple infectious complications, including pulmonary aspergillosis, co-infections by Stenotrophomonas maltophilia and COVID-19. Early initiation of supportive therapies and dexamethasone contributed to the control and resolution of these infections. This case reflects the importance of personalized decision-making and the potential benefits of CAR-T cell therapy in high-risk patients. Importantly, the advances made in the management of unforeseeable emergency conditions—such as the COVID-19 pandemic- now make it possible to ensure an adequate course of care for patients who are candidates for cell therapies (CAR-T/HSCT) where the time factor does not allow for postponement.

Finally, the Research Topic approached the impact of major complications on transplant outcomes, driving the reader into a manuscript able to provide a detailed and updated insight on GvHD and VOD.

Transplant outcome has progressively improved over time (5). However, GvHD remains one of the major life-threatening complications after allogeneic HSCT, mainly in case of severe and refractory forms (6, 7). The focus of the review by Gottardi et al. is the treatment of steroid refractory GvHD in children. In the pediatric setting, consolidated evidence regarding GvHD treatments is still lacking, and results are mainly derived from experiences on adult patients. Furthermore, the number of treated patients is limited, and the incidence of acute and chronic GvHD is lower, contributing to a very heterogeneous approach of this complication in this peculiar setting. Some therapies adopted in the adult setting have been evaluated also in children. Moreover, recently the increasing understanding of GvHD pathogenesis encouraged the adoption of targeted therapies and non-pharmacologic approaches, with higher response rates and lower immunosuppressive side effects. This Review critically explores the current experience on novel approaches to treat refractory GvHD within a pediatric setting.

Furthermore, the last article showcases a new screening approach to early identify patients at risk for hepatic VOD, also known as sinusoidal obstructive syndrome (SOS), a potentially life-threatening complication following HSCT procedure. During the decade, VOD/SOS was associated to decreased mortality rates, thanks to early intensive and multidisciplinary approaches (8). The brief research report from Avenoso et al. explores a new approach based on a focused VOD/SOS ward round, aiming to provide an early diagnosis of VOD/SOS, with prompt identification of patients at risk. In their experience they reported a rapid identification of patients with multiple risk factors for VOD/SOS, allowing an earlier diagnosis and the administration of specific treatment with defibrotide on the same day of diagnosis. Moreover, this approach may have high educational value for the health care professionals working within transplant services.

In conclusion, we hope that the readers appreciate the varied research included in this Research Topic, “Rising Stars in Cell and Stem Cell Transplantation”. The editorial team put substantial effort into screening and evaluating the high-quality manuscripts produced by researchers and their groups. We hope that this Research Topic will aid in the efforts to foster collaboration among researchers, as this is crucial element in the progression of research and science.
